# Comprehensive Analysis of 50 Edible Flowers From Yunnan Province: Active Components, Antioxidant Capacity, Tyrosinase Inhibition, and Antimicrobial Activity

**DOI:** 10.1002/fsn3.70666

**Published:** 2025-07-21

**Authors:** Yanxia Tao, Xingkai Zhang, Zhuangyu Li, Shouzheng Tian, Li Li, Xuhong Zhou

**Affiliations:** ^1^ School of Chinese Materia Medica Yunnan University of Chinese Medicine Kunming China; ^2^ Office of Science and Technology, Yunnan University of Chinese Medicine Kunming China

**Keywords:** anthocyanin, antimicrobial activity, antioxidant capacity, edible flowers, flavonoid, polyphenol, tyrosinase inhibition

## Abstract

Edible flowers are widely consumed for their potential health benefits. This study assessed the active components and in vitro antioxidant properties of 50 edible flowers from Yunnan to explore their potential uses. A 70% ethanol extract was prepared, and the total polyphenol content (TPC), total flavonoid content (TFC), and total anthocyanin content (TAC) were analyzed. Antioxidant capacity was evaluated using the ferric reducing antioxidant power (FRAP), 2,2‐diphenyl‐1‐picrylhydrazyl (DPPH), and 2,2′‐azino‐bis(3‐ethylbenzothiazoline‐6‐sulfonic acid) diammonium salt (ABTS) assays. Additionally, tyrosinase inhibition and antimicrobial activities were determined, and the correlation between active components and antioxidant capacity was investigated. The results showed that *Prunus mume* had high polyphenol content, *Osmanthus fragrans* Lour. had high flavonoid content, and *Rosa rugosa* Thunb. (Dianhong) had high anthocyanin content. *Rosa rugosa* Thunb. (Mohong) exhibited the strongest DPPH and ABTS radical scavenging activities. *Clerodendranthus spicatus* (Thunb.) C. Y. Wu demonstrated the highest FRAP activity, whereas *Punica granatum* L. showed the highest tyrosinase inhibition activity. *Nymphaea coerulea* exhibited significant antimicrobial effects against *Escherichia coli* and *Staphylococcus aureus* . Extracts from *Hibiscus rosa‐sinensis* and *Platycodon grandiflorus* (Jacq.) A. DC. demonstrated excellent antimicrobial effects against *Pseudomonas aeruginosa* . Correlation analysis indicated a positive relationship between TPC and antioxidant activities, suggesting that polyphenols are closely linked to antioxidant capacity. This study highlights the potential of these edible flowers as antimicrobial agents and natural antioxidants. Analysis using the membership function identified *N. coerulea* , *R. rugosa* , *P. granatum* , and *Lagerstroemia indica* L. as having the highest comprehensive scores, indicating their superior quality as sources of bioactive compounds.

## Graphical abstract

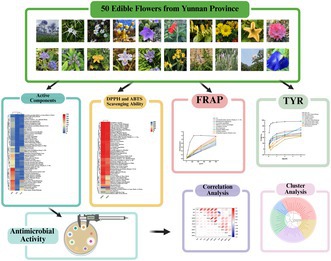

## 1 Introduction

Edible flowers refer to flowers and plants whose roots, leaves, and flowers can be consumed directly, offering a unique combination of nutritional and health benefits (Cong 2024), medicinal value, and beauty care properties (Purohit et al. 2021). These flowers not only serve as visual delights but also find application as ingredients in extracts, medicines, and the food industry, contributing significantly to economic value. Research indicates that certain edible flowers contain higher levels of vitamin C than some fruits, and some varieties even exceed the protein content of beef and eggs (Takahashi et al. 2020). In Yunnan Province, the abundance of plant resources has facilitated the rapid development of the edible flower industry since the late 20th century. Although the industry started relatively late, Yunnan now cultivates over 200 varieties of edible flowers across 42,000 ha, with 4.95 ha specifically designated for the province. Approximately 80% of the flower exports from the province consist of edible varieties, providing a solid foundation for flower‐based culinary innovations within the region (Wen et al. 2024).

Edible flowers are rich in protein, amino acids, fats, glucose, minerals, and other essential nutrients. They also contain various biologically active compounds (Zhao et al. 2024), such as polyphenols, flavonoids, and anthocyanins (Dujmović et al. 2022). Among these, the antioxidant properties of polyphenols and flavonoids have garnered significant attention in functional research on flowers. These compounds, secondary metabolites produced by plants in their natural environment, exhibit potent antioxidant capabilities when extracted (Loizzo et al. 2016). Flavonoids, a subclass of polyphenolic compounds, are commonly found in various parts of higher plants, including roots, stems, leaves, flowers, and fruits. They exhibit diverse structures and are present in abundant quantities (Behbahani et al. 2025). Research has demonstrated that flavonoids offer several health benefits, including protection against cardiovascular and cerebrovascular diseases, aging, inflammation, viruses, tumors, and pain, highlighting their vital role in promoting human health. Additionally, anthocyanins, which belong to the flavonoid category (Benvenuti et al. 2016), are widely distributed in flowering plants. These compounds significantly contribute to the color variations observed in flowers, as petal colors are primarily determined by the types of pigments present (Yue et al. 2024).

The antioxidant capacity of substances is often assessed using the ferric reducing antioxidant power (FRAP), 2,2‐diphenyl‐1‐picrylhydrazyl (DPPH), and 2,2′‐azino‐bis (3‐ethylbenzothiazoline‐6‐sulfonic acid) diammonium salt (ABTS) assays. These methods are essential for understanding the role of antioxidants in the pathogenesis and physiological processes of various diseases (Noshad, Behbahani, and Nikfarjam 2023). For instance, excessive levels of reactive oxygen species in the human body can induce toxicity and initiate a cascade of physiological and biochemical reactions that compromise cellular function and overall well‐being (Liu et al. 2025). Several ornamental and edible flowers have been identified as rich sources of natural antioxidants capable of neutralizing harmful free radicals (Zeng et al. 2014). These radicals are known to contribute to cellular damage, slow tissue degeneration, and accelerate aging‐related conditions such as heart disease, dementia, and tumors (Zhang, Shen, et al. 2023). Despite the benefits of natural antioxidants, most antioxidants currently used in food and cosmetic products are synthetic, and their long‐term use may pose potential health risks.

*Escherichia coli* is a common inhabitant of the intestinal tracts of humans and animals. Although most *E. coli* strains are non‐pathogenic and constitute a natural part of the intestinal flora (Jang et al. 2017), they are excreted in feces and released into the environment. However, under certain conditions, some strains can cause gastrointestinal or extraintestinal infections, other tissue and organ infections, such as urinary tract infections, in both humans and animals. These infections may result in symptoms such as gastroenteritis, diarrhea, abdominal pain, and nausea (Behbahani et al. 2024). *Staphylococcus aureus* , commonly referred to as “golden staph,” is a Gram‐positive bacterium that lacks spores and flagella; most strains also lack capsules. It is widely distributed in the environment, including air, water, dust, and human and animal excrement (Gu et al. 2022). Over time, *S. aureus* has emerged as a significant cause of complex infections in both hospitals and communities. It can lead to bacterial infections in humans and animals, ranging from mild skin and soft tissue infections to more severe conditions such as respiratory tract infections, pseudomembranous enteritis, and endocarditis (Tenover et al. 2006). In more serious cases, *S. aureus* may cause systemic infections such as bloodstream infections, septicemia, and toxic shock syndrome, posing serious risks to human health (Beltrán‐Martínez et al. 2024). Under favorable conditions, it can also produce enterotoxins that damage the intestines and lead to food poisoning. *Pseudomonas aeruginosa* is a Gram‐negative rod‐shaped bacterium and an opportunistic pathogen that infects both humans and animals (Riquelme et al. 2020). It presents significant health risks, particularly to elderly individuals or those with compromised immune systems. Common infections caused by *P. aeruginosa* include bacteremia, pneumonia, urinary tract infections, burn‐related infections, and secondary infections in cystic fibrosis patients (Jurado‐Martín et al. 2021). The emergence of drug‐resistant strains has elevated *P. aeruginosa* to one of the most serious nosocomial pathogens, posing substantial health risks. With the widespread and prolonged use of antibiotics, the severity of bacterial resistance has increased, leading to the evolution of superbugs and presenting critical threats to human health.

There is a growing demand for safe and effective antioxidants derived from natural and environmentally friendly sources, such as edible flowers (Guimarães et al. 2010). However, current research on edible flowers remains insufficient, highlighting the need to establish a more comprehensive foundation to facilitate their further investigation and application. This study investigated the levels of total polyphenols, total flavonoids, and anthocyanins in 50 varieties of edible flowers. Antioxidant capacity was evaluated using the DPPH and ABTS radical scavenging assays, as well as the FRAP method. Additionally, the study assessed tyrosinase (TYR) inhibition and antimicrobial activity in vitro, exploring the correlations between the levels of active compounds and antioxidant efficacy.

## 2 Materials and Methods

### 2.1 Flower Sample Collection

**FIGURE 1:**
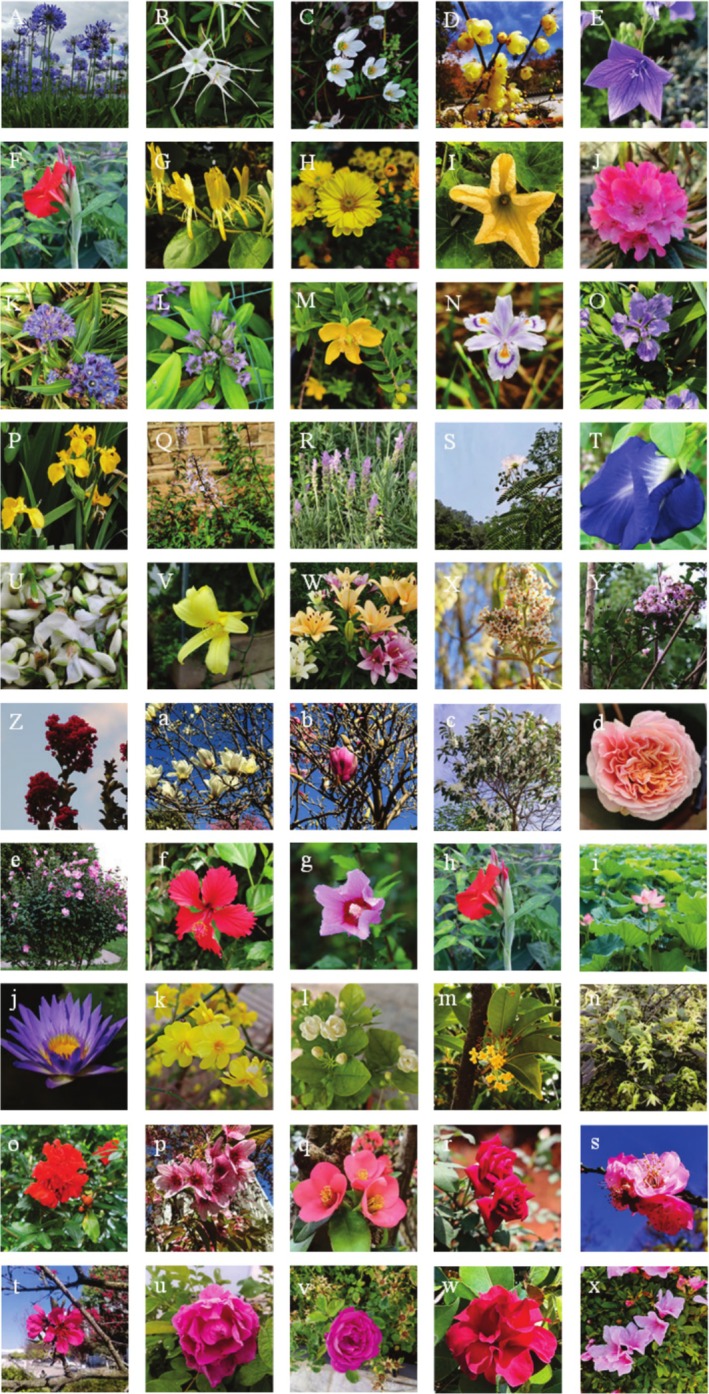
Information on 50 varieties of edible flowers. A, *Agapanthus africanus* (*Amaryllidaceae*); B, *Lycoris radiata* (L'Her.) Herb. (*Amaryllidaceae*); C, *Narcissus tazetta* L. var. *chinensis* Roem. (*Amaryllidaceae*); D, *Chimonanthus praecox* (Linn.) Link (*Calycanthaceae*); E, *Platycodon grandiflorus* (Jacq.) A. DC. (*Campanulaceae*); F, *Canna indica* L. (*Cannaceae*); G, *Lonicera japonica* Thunb. (*Caprifoliaceae*); H, *Dendranthema morifolium* (Ramat.) Tzvel. (*Compositae*); I, *Cucurbita moschata* (Duch. ex Lam.) Duch. ex Poiret (*Cucurbitaceae*); J, *Rhododendron lapponicum* (*Ericaceae*); K, *Gentiana macrophylla* Pall. (*Gentianaceae*); L, *Gentiana sino‐ornata* Balf. f. (*Gentianaceae*); M, *Hypericum monogynum* L. (*Guttiferae*); N, *Iridis Japonica* (*Iridaceae*); O, *Iris tectorum* (*Iridaceae*); P, *Iris pseudacorus* (*Iridaceae*); Q, *Clerodendranthus spicatus* (Thunb.) C. Y. Wu (*Labiatae*); R, *Lavandula angustifolia* (*Labiatae*); S, *Albizia julibrissin* Durazz. (*Leguminosae*); T, *Clitoria ternatea* (*Leguminosae*); U, *Sophora japonica* Linn. (*Leguminosae*); V, *Hemerocallis citrina* Baroni (*Liliaceae*); W, *Lilium formosanum* Wallace (*Liliaceae*); X, *Buddleja officinalis* Maxim. (*Loganiaceae*); Y, *Lagerstroemia indica* L. (Pink) (*Lythraceae*); Z, *Lagerstroemia indica* L. (Red) (*Lythraceae*); a, *Magnolia denudata* Desr. (White) (*Magnoliaceae*); b, *Magnolia denudata* Desr. (Purple) (*Magnoliaceae*); c, *Magnolia liliiflora* Desr (*Magnoliaceae*); d, *Althaea rosea* (Linn.) Cavan. (*Malvaceae*); e, *Hibiscus syriacus* Linn. (Double) (*Malvaceae*); f, *Hibiscus rosa‐sinensis* (*Malvaceae*); g, *Hibiscus syriacus* Linn. (Single) (*Malvaceae*); h, *Musa basjoo* (*Musaceae*); i, *Nelumbo nucifera* (*Nymphaeaceae*); j, *Nymphaea coerulea* (*Nymphaeaceae*); k, *Jasminum nudiflorum* Lindl. (*Oleaceae*); l, *Jasminum sambac* (L.) Ait. (*Oleaceae*); m, *Osmanthus fragrans* (Thunb.) Lour. (*Oleaceae*); n, *Dendrobium officinale* Kimura et Migo (*Orchidaceae*); o, *Punica granatum* L. (*Punicaceae*); p, *Cerasus serrulate* (*Rosaceae*); q, *Chaenomeles speciosa* (*Rosaceae*); r, *Rosa rugosa* Thunb. (Mohong) (*Rosaceae*); s, *Prunus mume* (*Rosaceae*); t, *Prunus persica* (*Rosaceae*); u, *Rosa damascene* (*Rosaceae*); v, *Rosa rugosa* Thunb. (Dianhong) (*Rosaceae*); w, *Camellia japonica* L. (Double) (*Theaceae*); x, *Camellia japonica* L. (Single) (*Theaceae*).

Fifty edible flower species from 25 families, primarily *Rosaceae*, *Magnoliaceae*, *Amaryllidaceae*, *Leguminosae*, *Malvaceae*, *Oleaceae*, and *Iridaceae*, were collected from Professor Xu‐hong Zhou's plantation in Kunming, Yunnan Province (Figure 1). The taxonomic classification of these flowers was based primarily on their morphological and structural characteristics. Species identification was conducted by Professor Xu‐hong Zhou, an expert in plant taxonomy.

### 2.2 Reagents and Apparatus

#### 2.2.1 Chemical Reagents

All reagents used were of analytical grade. Methanol, anhydrous ethanol, sodium hydroxide, anhydrous sodium carbonate, potassium persulfate, sodium nitrite, glacial acetic acid, FeSO_4_, FeCl_3_·6H_2_O, and aluminum trichloride were obtained from Tianjin Zhiyuan Chemical Reagent Co. Ltd. The DPPH radical was purchased from Phygene Life Sciences Co. Ltd. The Folin–Ciocalteu reagent, ABTS, and 2,4,6‐tri(2‐pyridyl)‐1,3,5‐triazine (TPTZ) were supplied by Macklin (Shanghai, China). Rutin, gallic acid, cyanidin‐3‐*O*‐glucoside, kojic acid, TYR, L‐DOPA, and tetracycline hydrochloride were purchased from Solarbio (Beijing, China). Sodium acetate trihydrate was purchased from Aladdin (Shanghai, China). L‐ascorbic acid (VC) was sourced from Guangdong Guanghua Technology Co. Ltd.

#### 2.2.2 Bacterial Strains and Culture Media

*E. coli* ATCC 25922, *S. aureus* ATCC 6538, and *P. aeruginosa* ATCC 9027 strains were purchased from Haibo Biotechnology Co. Ltd. (Qingdao High‐tech Industrial Park). Hydrolyzed casein peptone broth medium powder, Mueller‐Hinton agar medium powder, and nutrient agar medium were obtained from Guangdong Huankai Microbiology Technology Co. Ltd.

#### 2.2.3 Apparatus

The instruments used included an ultraviolet–visible spectrophotometer (UV‐9000, Hangzhou, China), an ultrasonic cleaner (SG3300HDT, Shanghai, China), a rotary evaporator (RV‐211A, Shanghai, China), a microplate reader (FlexA‐200, Hangzhou, China), an electro‐heating constant‐temperature incubator (BPX‐272, Hangzhou, China), a turbidity meter (ZC MCF, Beijing, China), a clean workstation (HCB‐1300V, Qingdao, China), and a vertical pressure steam sterilizer (SQ810C, Chongqing, China).

### 2.3 Extraction of Flower Components

Fresh petals stored at −80°C were first frozen with liquid nitrogen, then freeze‐dried and ground into a fine powder. Extraction was performed with 70% ethanol (1:25 w/v) under ultrasonic conditions at 60°C for 45 min. The extract was filtered, and ethanol was removed by rotary evaporation at 60°C. Final extracts were stored at −20°C for analysis.

### 2.4 Determination of Active Components

TPC was measured using the Folin–Ciocalteu colorimetric method following Dulf et al. (2016), with gallic acid as the standard to construct the calibration curve (*y* = 0.2207*x* − 0.01, *R* ^2^ = 0.9966). Results were expressed as milligrams of gallic acid equivalents per gram of sample (mg GAE/g). TFC was determined using a modified method on the basis of Dulf et al. (2015), with rutin as the standard (*y* = 0.8008*x* + 0.0385, *R* ^2^ = 0.9919), and results expressed as milligrams of rutin equivalents per gram of sample (mg RE/g). TAC was measured using a modified method on the basis of Li (2022), with cyanidin‐3‐*O*‐glucoside as the standard (*y* = 0.6668*x* − 0.0087, *R* ^2^ = 0.9986), and results expressed as milligrams of cyanidin‐3‐*O*‐glucoside equivalents per gram of sample (mg C3GE/g).

### 2.5 Determination of Antioxidant Activity

#### 2.5.1 Determination of DPPH Radical Scavenging Activity

The DPPH radical scavenging activity was determined using a modified method on the basis of Zhang et al. (2022). Vitamin C was used as the positive control. The DPPH scavenging rate was calculated using formula (1), and the IC_50_ value was determined using SPSS software.

(1) $$\text{DPPH scavenging rate}\;\left(\%\right)=\left[A-\left(B-C\right)/A\times 100\%\right]$$

where *A* represents the absorbance of the DPPH solution, *B* represents the absorbance of the sample mixed with the DPPH solution, and *C* represents the absorbance of the sample with methanol.

#### 2.5.2 Determination of ABTS Radical Scavenging Activity

The ABTS radical scavenging activity was measured using a modified method on the basis of Zhang et al. (2022). Vitamin C served as the positive control. The ABTS scavenging rate was calculated using formula (2), and the IC50 value was determined using SPSS software.

(2) $$\text{ABTS scavenging rate}\;\left(\%\right)=\left[A-\left(B-C\right)/A\times 100\%\right]$$

where *A* represents the absorbance of the ABTS solution, *B* represents the absorbance of the sample mixed with the ABTS solution, and *C* represents the absorbance of the sample mixed with absolute ethanol.

#### 2.5.3 Determination of FRAP

FRAP was evaluated using a modified method on the basis of Zhang, Cao, et al. (2023). A FeSO4 standard curve (*y* = 2.5588*x* − 0.0214, *R* ^2^ = 0.9994) was generated under the same conditions by replacing the sample with a FeSO4 solution. Absorbance values (*A*_*i*_ − *A*_*j*_) were applied to the standard curve using formula (3) to calculate total antioxidant activity.

(3) $$\text{FRAP}\left(\text{mmol}/L\right)=\left[\left(\mathrm{Ai}-\mathrm{Aj}\right)+0.0214\right]/2.5588$$

where *A*_*i*_ represents the absorbance of the test sample, and *A*_*j*_ represents the absorbance of the blank control group.

### 2.6 Determination of TYR Inhibition Activity

TYR inhibitory activity was assessed using a modified method on the basis of Yu et al. (2021). Briefly, 75 μL of the sample was mixed with 25 μL of TYR solution and incubated at 37°C for 10 min. Then, 100 μL of 1 mol/L L‐DOPA solution was added to initiate the reaction, and the absorbance was measured at 475 nm. Kojic acid served as the positive control. The TYR inhibition rate was calculated using formula (4).

(4) $$\text{Inhibition rate}\left(\%\right)=\left[\left(\mathrm{Ab}-A\right)-\left(\mathrm{Cb}-C\right)\right]/\left(\mathrm{Cb}-C\right)\times 100$$

where *A*_*b*_ represents the absorbance of the sample solution with TYR, A represents the absorbance of the sample solution with sodium phosphate buffer (pH 6.8), *C*_*b*_ represents the absorbance of the buffer (pH 6.8) with TYR, and *C* represents the absorbance of the buffer alone (pH 6.8).

### 2.7 Determination of In Vitro Antimicrobial Activity

#### 2.7.1 Preparation of Culture Medium and Bacterium Suspension

Hydrolyzed Casein Peptone Broth (21 g), Mueller‐Hinton Agar (38 g), and nutrient agar (33 g) powders were each dissolved in 1 L of distilled water by stirring and boiling (Shahab et al. 2023). Solutions were autoclaved at 121°C for 15 min, transferred into 500 mL flasks, labeled, and stored at 4°C.

Frozen *E. coli* , *S. aureus* , and *P. aeruginosa* were inoculated into Mueller‐Hinton Broth and incubated at 37°C, 125 rpm for 24 h (Mohammad et al. 2022). After centrifugation, bacterial pellets were resuspended in sterile saline to a concentration of 1.5 × 10^8^ CFU/mL. Suspensions were further diluted 1:1000 in sterile saline for minimum inhibitory concentration (MIC) determination.

#### 2.7.2 Determination of Bacteriostatic Circles

Antibacterial activity was assessed by the filter paper method (Noshad, Behbahani, Nikfarjam, and Zargari 2023). Sterile saline discs served as blank controls, and tetracycline was the positive control. Plates were incubated at 37°C for 24 h, after which inhibition zone diameters were measured. Sensitivity was interpreted on the basis of the inhibition zone diameter as follows: greater than 15 mm indicated high sensitivity; 10–15 mm, moderate sensitivity; 6–10 mm, low sensitivity; and less than 6 mm, no antibacterial effect.

#### 2.7.3 Determination of MIC and Minimum Bactericidal Concentration (MBC)

MIC refers to the lowest concentration of an antimicrobial agent required to prevent visible bacterial growth after overnight incubation. MIC values assess bacterial susceptibility to specific antimicrobial agents (Du et al. 2022). The MIC was determined using a previously described method (Ma et al. 2023).

Liquid culture medium (100 μL) showing no bacterial growth was transferred to agar plates and incubated at 37°C for 24 h. The bacterial colony count on the agar plates was determined using the viable count method (Mosallaie et al. 2024). The MBC is the lowest concentration of the test substance at which no bacterial growth, or fewer than five colonies, is observed on the plate.

### 2.8 Statistical Analysis

The experimental data were replicated three times, and the results were presented as mean ± standard deviation (SD). Statistical analysis was conducted using SPSS 27, applying one‐way ANOVA followed by Duncan's multiple range test for multiple comparisons. Statistical significance was set at *p* < 0.05. Additionally, correlation heatmap analysis and visualization were conducted using Origin 2022 software.

## 3 Results

### 3.1 Analysis of Active Components

**FIGURE 2:**
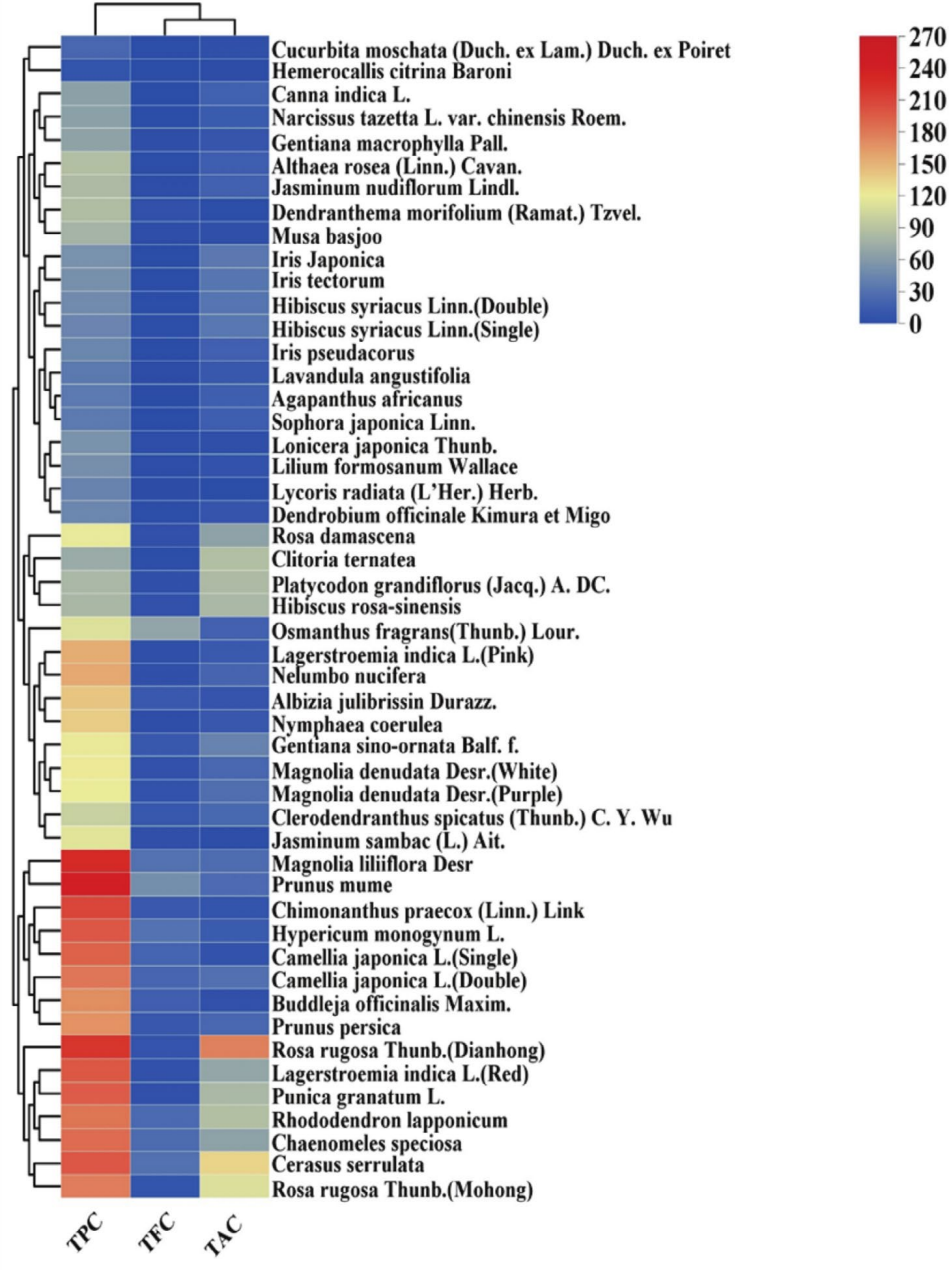
Heat map and cluster analysis of the active component contents of 50 edible flowers. TPC stands for the total polyphenols content, TFC stands for the total flavonoid content, and TAC stands for the anthocyanin content. The color transition of the rectangle from blue to red indicates an increase in active component contents.

Polyphenols, flavonoids, and anthocyanins are potent antioxidant components with significant free radical scavenging capacities. The active component contents of 50 edible flowers are shown in Figure 2. The edible flowers with the highest TPC were *P. mume, M. liliiflora*, and *R. rugosa* (Dianhong), with TPC values of 244.21 ± 1.73, 229.87 ± 9.45, and 221.67 ± 5.11 mg GAE/g, respectively. Conversely, *H. citrina and C. moschata* exhibited the lowest TPC values of 7.77 ± 0.11 and 23.31 ± 0.78 mg GAE/g, respectively. In addition, the edible flowers with the highest TFC were *O. fragrans*, *P. mume* , and *H. monogynum*, with TFC values of 68.93 ± 2.56 mg RE/g, 51.51 ± 8.20 mg RE/g, and 31.13 ± 1.75 mg RE/g, respectively. In contrast, *A. africanus and I. tectorum* had the lowest TFC values, at 0.02 ± 0.01 mg RE/g and 0.11 ± 0.02 mg RE/g, respectively. Moreover, the edible flowers with the highest TAC were *R. rugosa* (Dianhong), *C. serrulata*, and *R. rugosa* (Mohong), with TAC values of 177.42 ± 6.46, 134.92 ± 0.42, and 111.61 ± 3.25 mg C3GE/g, respectively. *D. morifolium* and *H. citrina* had the lowest TAC values, at 1.09 ± 0.04 and 1.59 ± 0.06 mg C3GE/g, respectively.

On the basis of these results, *P. mume* , *M. liliiflora*, and *R. rugosa* (Dianhong) were rich in polyphenols. *O. fragrans* , *P. mume* , and *H. monogynum* were abundant in flavonoids. *R. rugosa* (Dianhong), *C. serrulata* , and *R. rugosa* (Mohong) exhibited high anthocyanin content. Overall, the active components were abundant in *R. rugosa* (Dianhong), *P. mume* , *M. liliiflora*, *O. fragrans* , and *R. rugosa* (Mohong). Figure 2 illustrates the clustering of the 50 edible flowers into two main clusters and four sub‐clusters. Main cluster II comprised 15 edible flowers, including *R. rugosa* (Mohong), *C. serrulata* , *C. speciosa*, *R. lapponicum*, *P. granatum*, *L. indica*(Red), and *R. rugosa* (Dianhong), among others. The remaining 35 edible flowers formed main cluster I, which was divided into two sub‐clusters. Notably, *C. moschata* and *H. citrina* were grouped into the same subfamily.

### 3.2 Results of Antioxidant Capacity

#### 3.2.1 DPPH and ABTS Scavenging Ability Analysis

**FIGURE 3:**
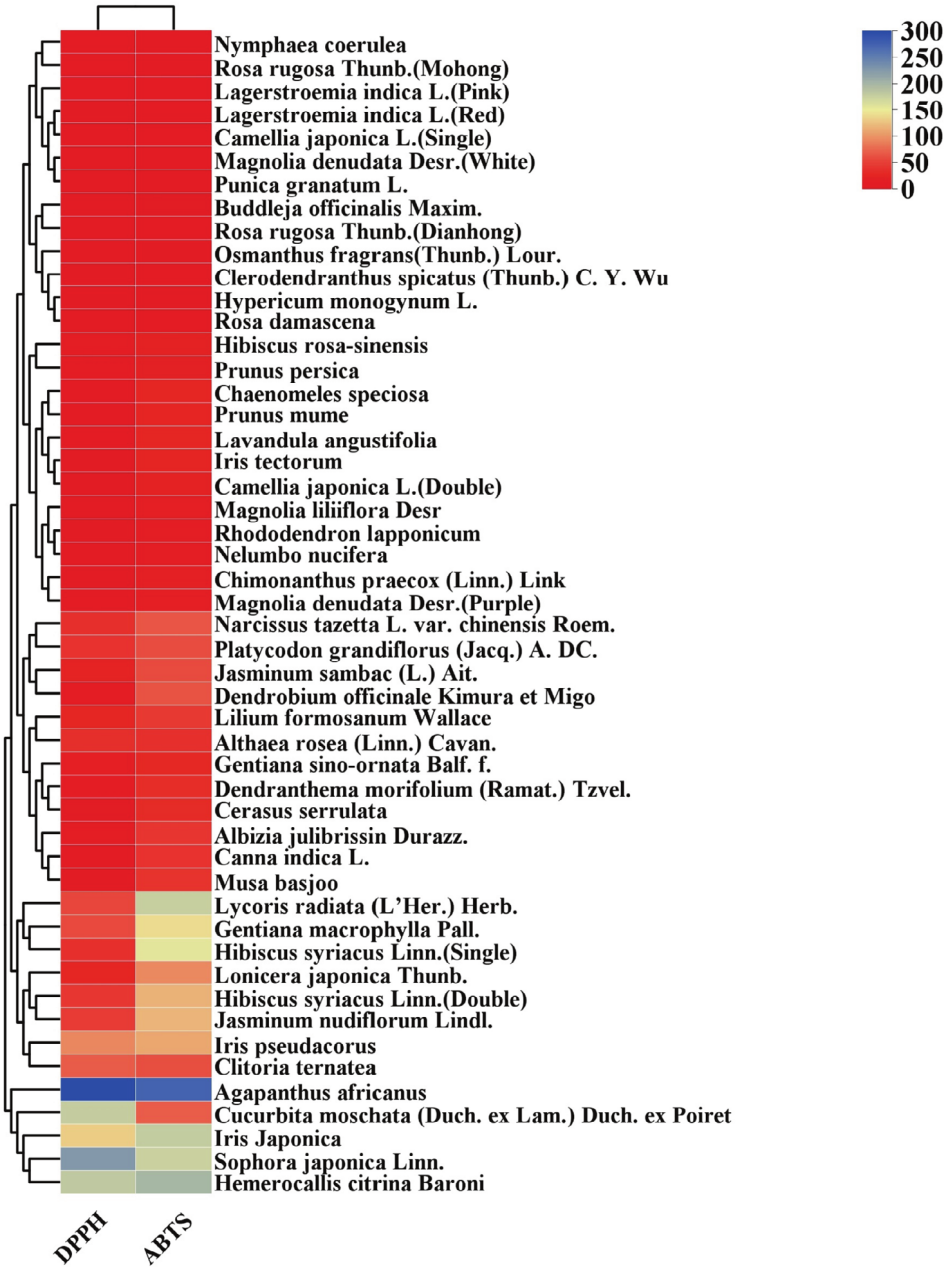
Heat map and cluster analysis of the DPPH and ABTS scavenging ability of 50 edible flowers. DPPH stands for DPPH scavenging ability, and ABTS stands for ABTS scavenging ability. The color transition of the rectangle from blue to red indicates an increase in scavenging ability.

The DPPH and ABTS radical scavenging assays are effective and sensitive methods for evaluating antioxidant capacity. These assays use IC_50_ values as key indicators of antioxidant strength, with lower IC_50_ values indicating higher antioxidant activity. The hierarchy of ABTS radical scavenging activity correlated with that of DPPH radical scavenging capacity on the basis of their IC_50_ values (Shi et al. 2009). The antioxidant activity results of 50 edible flowers are shown in Figure 3.

In the DPPH radical scavenging assay, VC was used as a positive control, with an IC_50_ value of 0.62 ± 0.03 μg/mL. *R. rugosa* (Mohong) showed the highest DPPH radical scavenging activity among the 50 edible flowers, with an IC_50_ value of 0.94 ± 0.03 μg/mL. *N. coerulea*, *L. indica* (Pink), *L. indica* (Red), and *M. denudata* (White) also showed strong DPPH radical scavenging ability, with IC_50_ values of 1.12 ± 0.10, 1.35 ± 0.04, 2.26 ± 0.08, and 2.49 ± 0.17 μg/mL, respectively. Conversely, *A. africanus* , *S. japonica*, and *H. citrina* exhibited the weakest DPPH radical scavenging capacities, with IC_50_ values of 299.91 ± 57.29, 227.95 ± 3.21, and 182.78 ± 19.11 μg/mL, respectively.

In the ABTS radical scavenging assay, VC also served as the positive control, with an IC_50_ value of 2.26 ± 0.15 μg/mL. *R. rugosa* (Mohong) showed the highest ABTS radical scavenging activity, with an IC_50_ value of 6.04 ± 0.10 μg/mL. *M. denudata* (White), *P. granatum* , *N. coerulea* , and *L. indica* (Red) also showed high ABTS radical scavenging activity, with IC_50_ values of 6.72 ± 0.21, 7.16 ± 0.19, 7.29 ± 0.39, and 7.72 ± 0.78 μg/mL, respectively. On the other hand, *A. africanus* , *H. citrina* , and *H. iridis* exhibited the weakest ABTS radical scavenging capacities, with IC_50_ values of 274.89 ± 13.78, 200.03 ± 35.92, and 180.70 ± 1.69 μg/mL, respectively.

The findings highlight that *R. rugosa* (Mohong), *M. denudata* (White), *N. coerulea* , and *L. indica* (Red) had the highest antioxidant scavenging capacities. As shown in Figure 3, the 50 edible flowers were grouped into two main clusters. Edible flowers with the highest antioxidant capacities, such as *N. coerulea* , *R. rugosa* (Mohong), *L. indica* (Pink), *L. indica* (Red), *Camellia japonica* (Single), *M. denudata* (White), and *P. granatum* , were classified into the same subfamily group. Meanwhile, edible flowers with the weakest antioxidant capacities, including *A. africanus* , *C. moschata* , *H. iridis* , *S. japonica* , and *H. citrina* , formed part of main cluster II.

#### 3.2.2 FRAP Analysis

**FIGURE 4:**
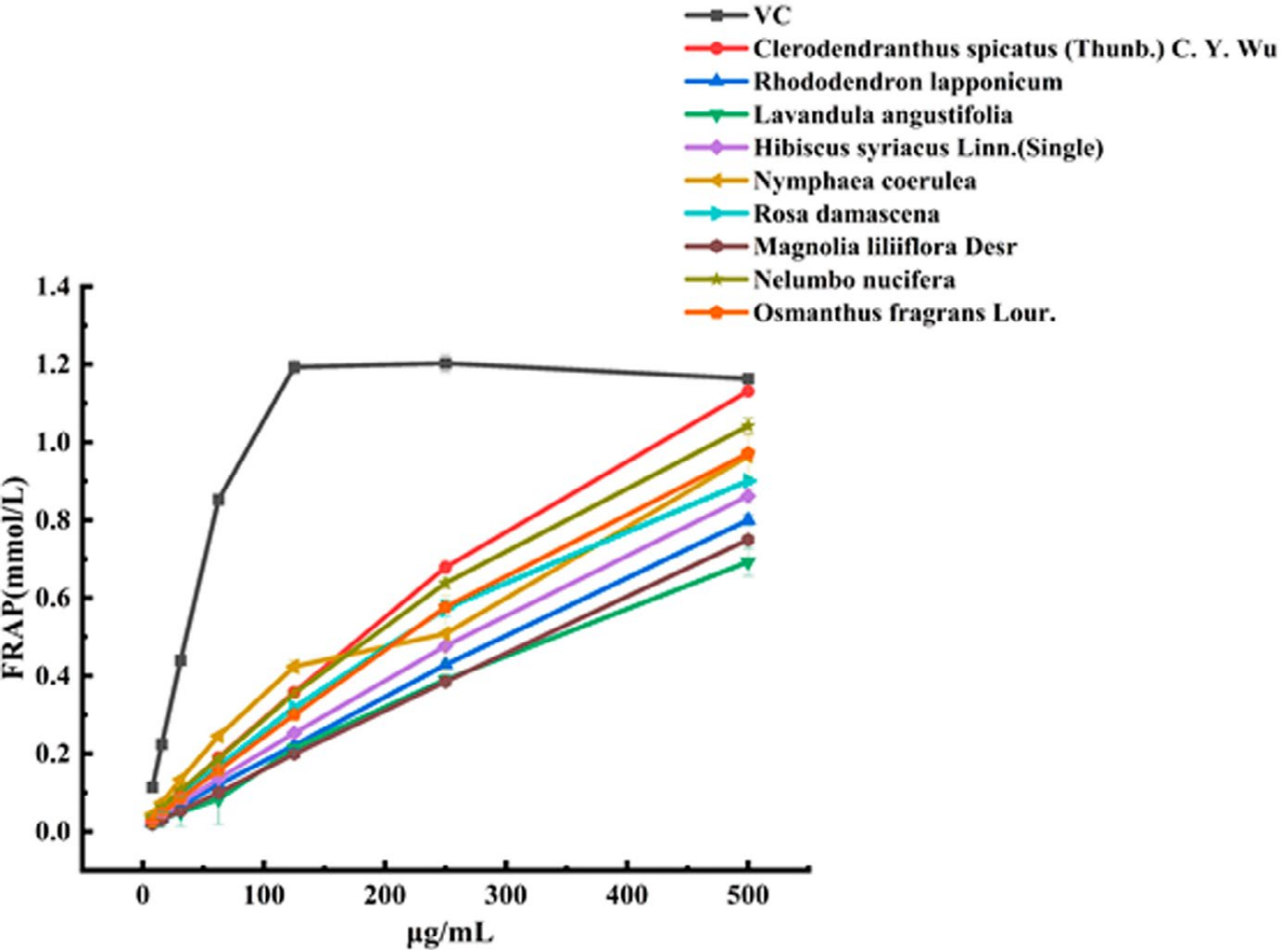
Ferric reducing antioxidant power (FRAP) of 9 edible flowers with the strongest FRAP among the 50 edible flowers. FRAP stands for ferric reducing antioxidant power. Vitamin C was used as a positive control.

The FRAP assay measures antioxidant capacity by reducing Fe^3+^‐TPTZ to Fe^2+^‐TPTZ (Kaisoon et al. 2011). The FRAP results for the 50 edible flowers are shown in Table 1. The nine edible flowers with the highest FRAP values were selected for further analysis, using VC as a positive control. The results indicated that *C. spicatus* exhibited the strongest FRAP among the 50 edible flowers, followed by *N. nucifera*, *O. fragrans* , and *N. coerulea* . *L. angustifolia* and *M. liliiflora* showed relatively weaker FRAP values among the top nine flowers (Figure 4). Comprehensive data are presented in Table S.

**TABLE 1:** Ferric reducing antioxidant power and tyrosinase inhibition activity of the 50 edible flowers.

No.	Flower samples	FRAP	TYR
1	*Agapanthus africanus*	+	+
2	*Lycoris radiata* (L'Her.) Herb.	+	−
3	*Narcissus tazetta* L. var. chinensis Roem.	+	+
4	*Chimonanthus praecox* (Linn.) Link	+	+
5	*Platycodon grandiflorus* (Jacq.) A. DC.	+	+
6	*Canna indica* L.	+	−
7	*Lonicera japonica* Thunb.	+	+
8	*Dendranthema morifolium* (Ramat.) Tzvel.	+	+
9	*Cucurbita moschata* (Duch. ex Lam.) Duch. ex Poiret	+	+
10	*Rhododendron lapponicum*	+	+
11	*Gentiana macrophylla* Pall.	+	+
12	*Gentiana sino‐ornata* Balf. f.	+	+
13	*Hypericum monogynum* L.	+	−
14	*Iridis Japonica*	+	+
15	*Iris tectorum*	+	+
16	*Iris pseudacorus*	+	+
17	*Clerodendranthus spicatus* (Thunb.) C. Y. Wu	+	−
18	*Lavandula angustifolia*	+	−
19	*Albizia julibrissin* Durazz.	+	+
20	*Clitoria ternatea*	+	+
21	*Sophora japonica* Linn.	+	+
22	*Hemerocallis citrina* Baroni	+	−
23	*Lilium formosanum* Wallace	+	+
24	*Buddleja officinalis* Maxim.	+	−
25	*Lagerstroemia indica* L. (Pink)	+	+
26	*Lagerstroemia indica* L. (Red)	+	+
27	*Magnolia denudata* Desr. (White)	+	−
28	*Magnolia denudata* Desr. (Purple)	+	−
29	*Magnolia liliiflora* Desr	+	−
30	*Althaea rosea* (Linn.) Cavan.	+	−
31	*Hibiscus syriacus* Linn. (Double)	+	+
32	*Hibiscus rosa‐sinensis*	+	−
33	*Hibiscus syriacus* Linn. (Single)	+	+
34	*Musa basjoo*	+	+
35	*Nelumbo nucifera*	+	+
36	*Nymphaea coerulea*	+	+
37	*Jasminum nudiflorum* Lindl.	+	+
38	*Jasminum sambac* (L.) Ait.	+	+
39	*Osmanthus fragrans* (Thunb.) Lour.	+	−
40	*Dendrobium officinale* Kimura et Migo	+	+
41	*Punica granatum* L.	+	+
42	*Cerasus serrulata*	+	+
43	*Chaenomeles speciosa*	+	−
44	*Rosa rugosa* Thunb. (Mohong)	+	+
45	*Prunus mume*	+	−
46	*Prunus persica*	+	−
47	*Rosa damascena*	+	+
48	*Rosa rugosa* Thunb. (Dianhong)	+	+
49	*Camellia japonica* L. (Double)	+	−
50	*Camellia japonica* L. (Single)	+	−

*Note:* “+” indicates that the edible flowers exhibit ferric reducing antioxidant power or tyrosinase inhibition activity, whereas “−” indicates that they do not.

### 3.3 Tyrosinase Inhibition Activity Analysis

**FIGURE 5:**
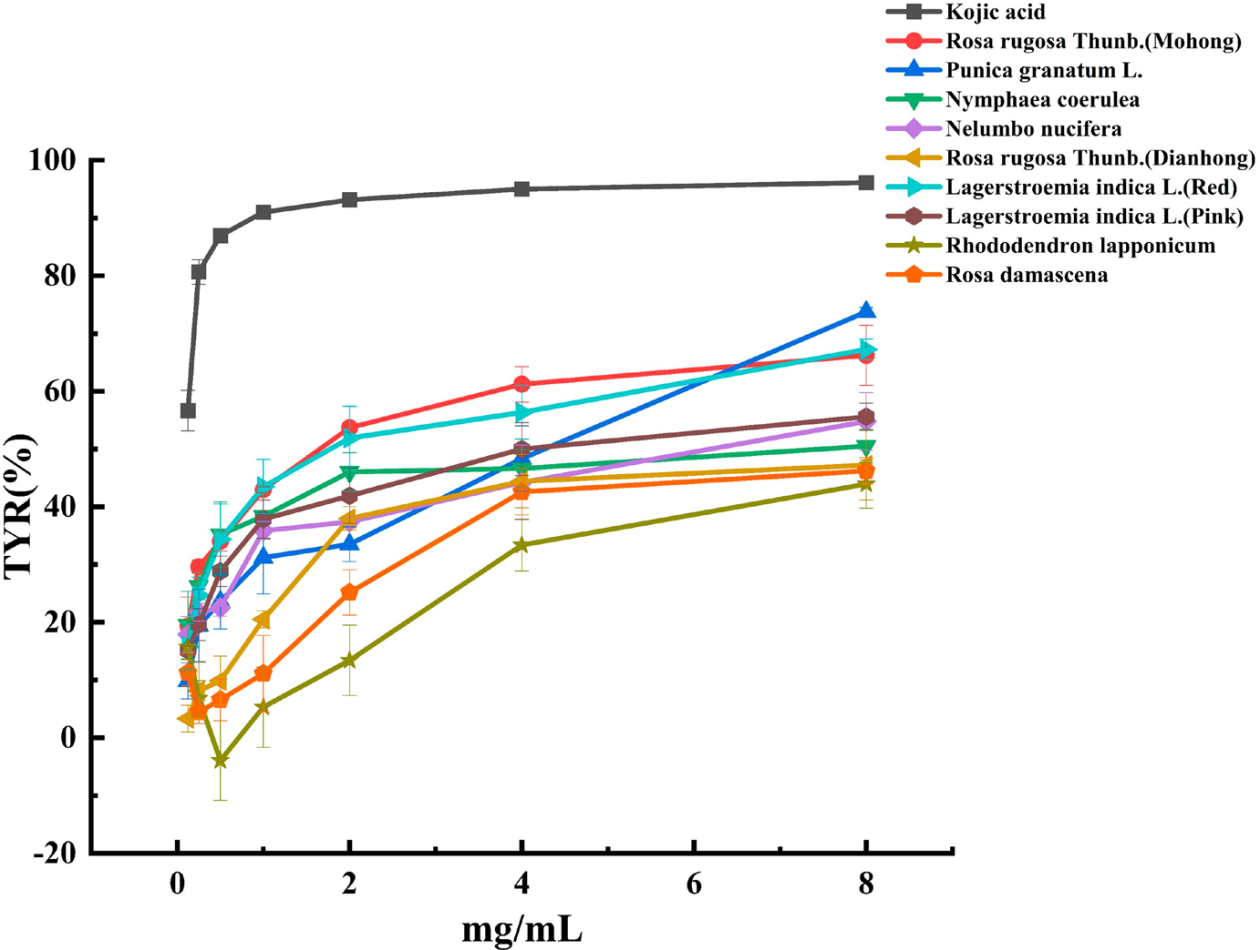
Tyrosinase inhibition activity of 9 edible flowers with the strongest TYR inhibition activity among the 50 edible flowers. TYR stands for tyrosinase inhibition activity. Kojic acid was used as a positive control.

TYR is a key enzyme that regulates melanin production, acting as the rate‐limiting factor in this process. TYR inhibitors can effectively modulate the overproduction of melanin by hindering TYR activity, making them a promising treatment approach for pigmentation disorders (Solimine et al. 2016). The TYR inhibition activity results for 50 edible flowers are shown in Table 1. Nine edible flowers with the highest TYR inhibition activity were selected for illustration, using kojic acid as a positive control. The results showed that *P. granatum* exhibited the highest TYR inhibition activity among the 50 edible flowers, followed by *L. indica* (Red), *R. rugosa* (Mohong), and *L. indica* (Pink). In contrast, *R. lapponicum* and *R. damascene* had weaker TYR inhibition activity among the top nine edible flowers (Figure 5). Several edible flowers did not demonstrate TYR inhibition activity, including *L. radiata*, *C. indica*, *L. angustifolia* , *H. rosa‐sinensis*, and *P. mume* . Comprehensive data are presented in Table S.

### 3.4 In Vitro Antimicrobial Activity

#### 3.4.1 Antimicrobial Activity Analysis

The antimicrobial activity of extracts from 50 edible flowers is presented in Table 2, with tetracycline hydrochloride used as a positive control. Among the 50 edible flowers, 36 exhibited varying degrees of inhibitory activity against *E. coli* . Extracts from *N. coerulea* , *L. indica* (Red), *P. granatum* , *L. japonica*, and *L. indica* (Pink) exhibited significant antimicrobial activity against *E. coli* , with inhibition zones ranging from 10 to 15 mm, indicating moderate sensitivity. Extracts from 31 edible flowers, including *L. formosanum*, *I. japonica*, *N. nucifera* , *I. tectorum* , and *C. japonica* (Single), exhibited antimicrobial activity against *E. coli* , with inhibition zones ranging from 6 to 10 mm, indicating low sensitivity. In contrast, extracts from 14 edible flowers, such as *G. sino‐ornata*, *M. liliiflora*, and *B. officinalis*, showed no inhibitory effects against *E. coli* .

**TABLE 2:** Determination results of in vitro bacteriostatic activity of the 50 edible flowers.

Samples	The bacteriostatic diameter (mm)		
	*E. coli*	*S. aureus*	*P. aeruginosa*
Tetracycline hydrochloride	29.68 ± 0.50	28.71 ± 1.48	30.08 ± 0.24
*Agapanthus africanus*	—	—	—
*Lycoris radiata* (L'Her.) Herb.	7.66 ± 0.25	12.30 ± 2.49	—
*Narcissus tazetta* L. var. chinensis Roem.	6.33 ± 0.05	7.09 ± 0.05	—
*Chimonanthus praecox* (Linn.) Link	—	—	—
*Platycodon grandiflorus* (Jacq.) A. DC.	6.50 ± 0.01	7.04 ± 0.07	15.74 ± 1.09
*Canna indica* L.	8.12 ± 0.11	8.31 ± 0.70	—
*Lonicera japonica* Thunb.	10.38 ± 0.54	9.95 ± 0.52	6.50 ± 0.10
*Dendranthema morifolium* (Ramat.) Tzvel.	8.43 ± 0.09	8.75 ± 0.75	7.00 ± 1.20
*Cucurbita moschata* (Duch. ex Lam.) Duch. ex Poiret	6.34 ± 0.19	6.21 ± 0.02	8.55 ± 0.62
*Rhododendron lapponicum*	8.71 ± 0.50	7.20 ± 0.28	8.63 ± 1.23
*Gentiana macrophylla* Pall.	6.67 ± 0.43	7.04 ± 0.08	—
*Gentiana sino‐ornata* Balf. f.	—	9.99 ± 1.75	—
*Hypericum monogynum* L.	—	7.42 ± 0.53	10.67 ± 0.47
*Iridis Japonica*	9.91 ± 0.19	6.25 ± 0.05	14.40 ± 0.64
*Iris tectorum*	9.35 ± 0.49	—	8.08 ± 0.14
*Iris pseudacorus*	—	—	—
*Clerodendranthus spicatus* (Thunb.) C. Y. Wu	—	—	7.00 ± 0.02
*Lavandula angustifolia*	6.58 ± 0.40	7.60 ± 0.50	6.50 ± 0.05
*Albizia julibrissin* Durazz.	9.14 ± 0.10	6.41 ± 0.02	7.00 ± 0.40
*Clitoria ternatea*	6.49 ± 0.20	8.15 ± 0.24	10.06 ± 0.07
*Sophora japonica* Linn.	6.16 ± 0.07	8.32 ± 0.76	11.34 ± 2.30
*Hemerocallis citrina* Baroni	—	—	6.49 ± 0.45
*Lilium formosanum* Wallace	9.97 ± 0.05	6.54 ± 0.07	9.60 ± 0.57
*Buddleja officinalis* Maxim.	—	7.51 ± 0.38	9.39 ± 1.25
*Lagerstroemia indica* L. (Pink)	11.17 ± 0.29	11.93 ± 0.90	13.00 ± 0.50
*Lagerstroemia indica* L. (Red)	10.17 ± 0.76	10.90 ± 0.79	11.50 ± 0.50
*Magnolia denudata* Desr. (White)	8.27 ± 0.64	—	7.33 ± 0.29
*Magnolia denudata* Desr. (Purple)	—	9.39 ± 0.43	8.96 ± 0.27
*Magnolia liliiflora* Desr	—	6.49 ± 0.01	7.00 ± 0.50
*Althaea rosea* (Linn.) Cavan.	8.54 ± 0.36	—	—
*Hibiscus syriacus* Linn. (Double)	8.55 ± 0.66	6.50 ± 0.00	7.46 ± 0.33
*Hibiscus rosa‐sinensis*	7.82 ± 0.14	—	16.20 ± 1.04
*Hibiscus syriacus* Linn. (Single)	—	—	—
*Musa basjoo*	7.50 ± 0.00	—	8.23 ± 0.25
*Nelumbo nucifera*	9.56 ± 0.32	8.63 ± 0.09	8.03 ± 0.06
*Nymphaea coerulea*	12.11 ± 0.85	12.42 ± 0.61	7.00 ± 0.10
*Jasminum nudiflorum* Lindl.	8.50 ± 0.87	9.13 ± 0.32	8.40 ± 0.17
*Jasminum sambac* (L.) Ait.	6.27 ± 0.12	9.19 ± 0.98	6.70 ± 0.20
*Osmanthus fragrans* (Thunb.) Lour.	7.80 ± 0.26	10.23 ± 0.25	7.50 ± 0.00
*Dendrobium officinale* Kimura et Migo	8.97 ± 0.06	8.97 ± 0.06	7.00 ± 0.00
*Punica granatum* L.	10.97 ± 0.98	10.88 ± 0.24	10.77 ± 0.11
*Cerasus serrulata*	8.25 ± 1.11	—	—
*Chaenomeles speciosa*	6.61 ± 0.25	9.33 ± 0.28	6.42 ± 0.08
*Rosa rugosa* Thunb. (Mohong)	—	10.33 ± 0.60	6.72 ± 0.51
*Prunus mume*	8.63 ± 0.44	7.45 ± 2.10	8.12 ± 0.13
*Prunus persica*	—	10.16 ± 2.27	7.97 ± 0.07
*Rosa damascena*	9.06 ± 0.20	8.04 ± 0.46	—
*Rosa rugosa* Thunb. (Dianhong)	—	8.10 ± 0.06	8.63 ± 0.10
*Camellia japonica* L. (Double)	9.07 ± 0.12	—	8.00 ± 0.00
*Camellia japonica* L. (Single)	9.25 ± 0.35	—	7.67 ± 0.29

*Note:* Values are shown as the mean ± SD (*n* = 3). “—” indicates that the edible flowers do not exhibit bacteriostatic activity.

As shown in Table 2, 36 edible flowers exhibited varying levels of inhibitory effects against *S. aureus* . Extracts from 8 flowers, including *N. coerulea* , *L. radiata* , *L. indica* (Pink), *L. indica* (Red), and *P. granatum* , exhibited significant antimicrobial effects, with inhibition zones ranging from 10 to 15 mm, indicating moderate sensitivity. Extracts from 28 edible flowers, including *G. sino‐ornata*, *L. japonica* , *M. denudata* (Purple), *C. speciosa* , and *J. sambac*, exhibited antimicrobial activity with inhibition zones of 6 to 10 mm, indicating low sensitivity. However, extracts from 14 edible flowers, including *I. tectorum* , *C. serrulata* , and *M. basjoo*, exhibited no antimicrobial activity against *S. aureus* .

As shown in Table 2, 38 edible flowers exhibited varying inhibitory effects against *P. aeruginosa* . Extracts from *H. rosa‐sinensis* and *P. grandiflorus* exhibited excellent antimicrobial activity, with inhibition zones higher than 15 mm, indicating high sensitivity. Extracts from 7 edible flowers, including *I. japonica* , *L. indica* (Pink), *L. indica* (Red), *S. japonica* , and *P. granatum* , exhibited significant antimicrobial activity, with inhibition zones of 10 to 15 mm, indicating moderate sensitivity. Extracts from 29 edible flowers, including *L. formosanum* , *B. officinalis* , *M. denudata* (Purple), *R. rugosa* (Dianhong), and *R. lapponicum* , exhibited antimicrobial activity, with inhibition zones ranging from 6 to 10 mm, indicating low sensitivity. In contrast, extracts from 12 edible flowers, including *R. damascena*, *A. rosea*, and *C. serrulata* , exhibited no antimicrobial effects against *P. aeruginosa* .

#### 3.4.2 MIC and MBC Analyses

The MIC and MBC values of different edible flower extracts against *E. coli* are shown in Table 3. For *N. coerulea*, the MIC and MBC were 0.6250 mg/mL and 1.2500 mg/mL, respectively. *L. indica* (Pink) and *P. granatum* exhibited MIC and MBC values of 0.3125 mg/mL and 0.6250 mg/mL, respectively. *L. japonica* had MIC and MBC values of 1.2500 mg/mL and 2.5000 mg/mL, respectively. *L. indica* (Red) showed MIC and MBC values of 0.3125 mg/mL and 1.2500 mg/mL. These results showed that *L. indica* (Pink) and *P. granatum* had the highest inhibitory effects against *E. coli* , followed by *L. indica* (Red), *N. coerulea* , and *L. japonica* .

**TABLE 3:** Determination results of MIC and MBC of five edible flower extracts against *E. coli* .

Flower samples	MIC (mg/mL)	MBC (mg/mL)
*Nymphaea coerulea*	0.6250	1.2500
*Punica granatum* L.	0.3125	0.6250
*Lonicera japonica* Thunb.	1.2500	2.5000
*Lagerstroemia indica* L. (Red)	0.3125	1.2500
*Lagerstroemia indica* L. (Pink)	0.3125	0.6250

The MIC and MBC values of edible flower extracts against *S. aureus* are shown in Table 4. *N. coerulea* exhibited MIC and MBC values of 0.6250 mg/mL and 1.2500 mg/mL, respectively. In contrast, *P. granatum* showed values of 0.3125 mg/mL and 2.5000 mg/mL, respectively. *R. rugosa* (Mohong) had MIC and MBC values of 0.0781 mg/mL and 1.2500 mg/mL, respectively. *L. indica* (Red) and *L. indica* (Pink) exhibited MIC values of 0.3125 mg/mL, with MBC values of 0.6250 mg/mL and 1.2500 mg/mL, respectively. *L. radiata* showed an MIC of 2.5000 mg/mL and an MBC exceeding 2.5000 mg/mL. Both *P. persica* and *O. fragrans* showed MIC values of 1.2500 mg/mL and MBC values greater than 2.5000 mg/mL. These results showed that *R. rugosa* (Mohong) exhibited the highest inhibitory effect against *S. aureus* , followed by *L. indica* (Red), *L. indica* (Pink), *P. granatum* , *N. coerulea* , *P. persica* , *O. fragrans* , and *L. radiata* .

**TABLE 4:** Determination results of MIC and MBC of eight edible flower extracts against *S. aureus* .

Flower samples	MIC (mg/mL)	MBC (mg/mL)
*Nymphaea coerulea*	0.6250	1.2500
*Lycoris radiata* (L'Her.) Herb	2.5000	> 2.5000
*Punica granatum* L.	0.3125	2.5000
*Rosa rugosa* Thunb. (Mohong)	0.0781	1.2500
*Prunus persica*	1.2500	> 2.5000
*Lagerstroemia indica* L. (Red)	0.3125	0.6250
*Lagerstroemia indica* L. (Pink)	0.3125	1.2500
*Osmanthus fragrans* (Thunb.) Lour.	1.2500	> 2.5000

The MIC and MBC values of edible flower extracts against *P. aeruginosa* are shown in Table 5. *H. rosa‐sinensis* exhibited MIC and MBC values of 1.2500 mg/mL and 2.5000 mg/mL, respectively. *S. japonica* had MIC and MBC values of 0.6250 mg/mL and 1.2500 mg/mL, respectively. Both *P. granatum* and *H. monogynum* exhibited MIC values of 0.3125 mg/mL and MBC values of 2.5000 mg/mL. *L. indica* (Pink) exhibited MIC and MBC values of 0.1562 mg/mL and 0.3125 mg/mL, respectively, whereas *L. indica* (Red) showed values of 0.3125 mg/mL and 0.6250 mg/mL. *P. grandiflorus*, *I. japonica* , and *Clitoria ternatea* had MIC values of 2.5000 mg/mL, 0.6250 mg/mL, and 0.3125 mg/mL, respectively, with MBC values exceeding 2.5000 mg/mL. Among all tested flowers, *L. indica* (Pink) demonstrated the highest inhibitory effect against *P. aeruginosa* , followed by *L. indica* (Red), *P. granatum* , *H. monogynum*, *C. ternatea* , *S. japonica* , *I. japonica* , *H. rosa‐sinensis* , and *P. grandiflorus* .

**TABLE 5:** Determination results of MIC and MBC of nine edible flower extracts against *P. aeruginosa* .

Flower samples	MIC (mg/mL)	MBC (mg/mL)
*Hibiscus rosa‐sinensis*	1.2500	2.5000
*Platycodon grandiflorus* (Jacq.) A. DC.	2.5000	> 2.5000
*Iris japonica*	0.6250	> 2.5000
*Sophora japonica* Linn.	0.6250	1.2500
*Punica granatum* L.	0.3125	2.5000
*Hypericum monogynum* L.	0.3125	2.5000
*Clitoria ternatea*	0.3125	> 2.5000
*Lagerstroemia indica* L. (Pink)	0.1562	0.3125
*Lagerstroemia indica* L. (Red)	0.3125	0.6250

### 3.5 Comprehensive Evaluation on the Basis of Flower Active Components and Antioxidant Capacity

#### 3.5.1 Correlation Analysis

**FIGURE 6:**
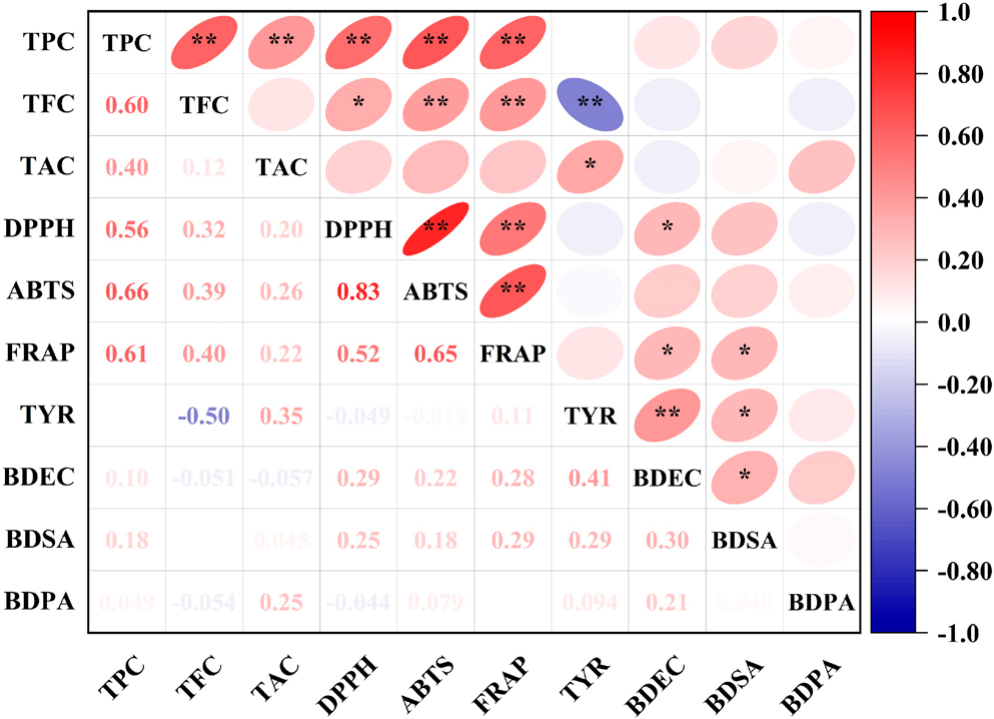
Pearson correlation analysis of active components, antioxidant indices, tyrosinase inhibition activity, and bacteriostatic activity of the 50 edible flowers. Ellipse in color indicates the correlation between two indicators distributed on the vertical and horizontal axes, respectively. The transition in the shape and color of the ellipse from round to flat and from blue to red indicates increasing correlation strenghth. * and ** denote significant correlation at *p* < 0.05 and *p* < 0.01 levels, respectively. BDEC stands for the bacteriostatic diameter of *E. coli*, BDSA for *S. aureus*, BDPA for *P. aeruginosa* .

The relationships among active components, antioxidant indices, TYR inhibition activity, and antimicrobial activity in 50 edible flowers are shown in Figure 6. The correlation analysis revealed that TPC was significantly (*p* < 0.05) positively correlated with FRAP values, DPPH scavenging rates, and particularly ABTS scavenging rates, with a correlation coefficient of 0.66. A strong correlation between ABTS radical scavenging activity and TPC indicates that the antioxidant capacity of edible flowers is closely linked to their polyphenol content. For instance, flowers with higher polyphenol content, such as *R. rugosa* (Dianhong), demonstrated superior antioxidant capacity among the 50 edible flowers investigated.

#### 3.5.2 Cluster Analysis

**FIGURE 7:**
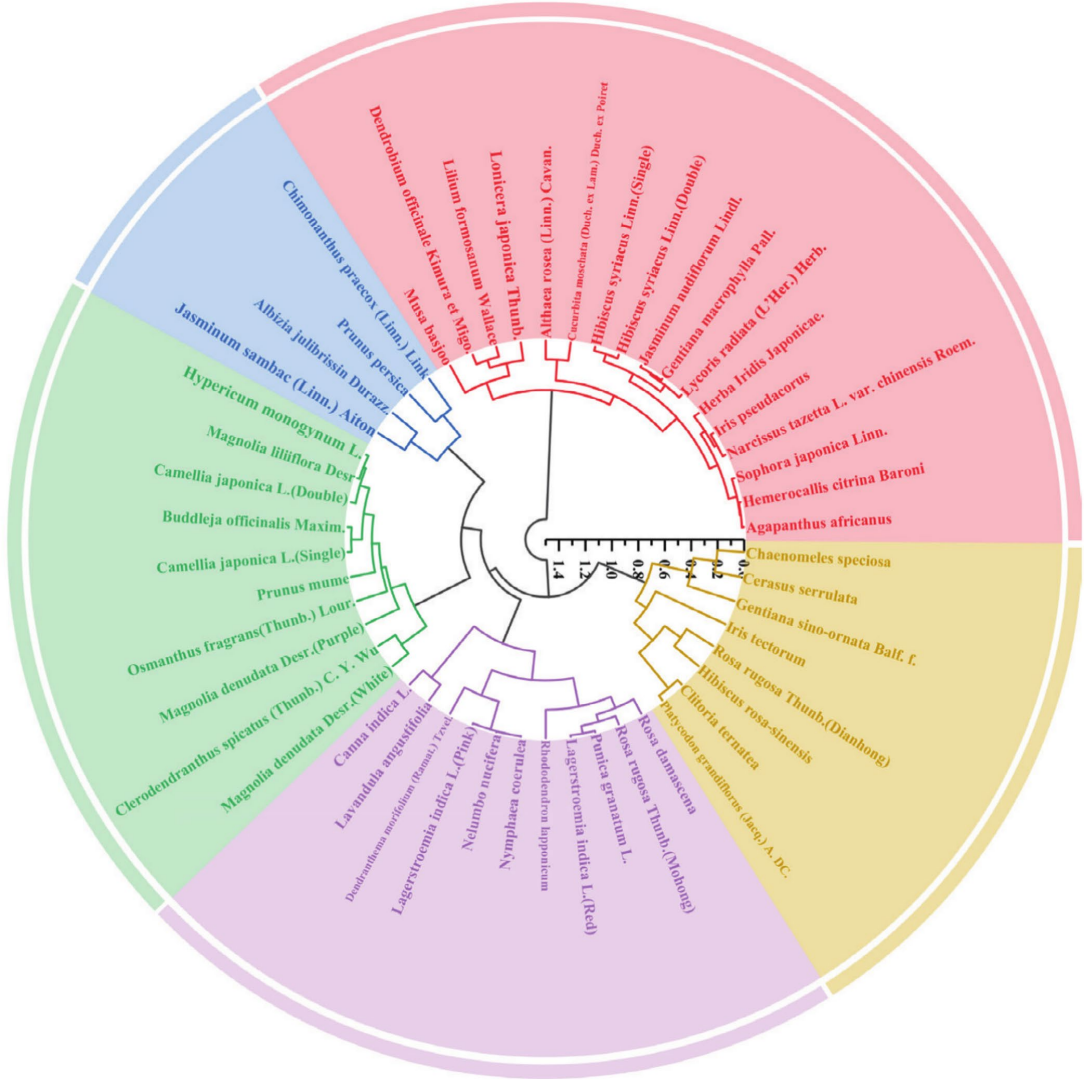
Dendrogram showing clustering pattern of 50 edible flowers on the basis of 10 indices.

A dendrogram is a visual tool that represents the similarity or dissimilarity between different objects, often expressed as distance relationships. Hierarchical clustering and dendrograms are specifically employed to evaluate overall distances in expression relationships among samples. The proximity of leaves in the dendrogram reflects their grouping into clusters, whereas the height of each branch indicates the degree of similarity or dissimilarity between clusters, with greater heights suggesting higher disparity between pairs of leaves (Dhiman et al. 2017). For this analysis, 10 indices affecting sample quality were selected, including total polyphenols, total flavonoids, anthocyanin content, FRAP values, DPPH and ABTS radical scavenging rates, TYR inhibition activity, and antimicrobial activity, as shown in Figure 7. The results indicated that the 50 edible flowers were divided into 5 main clusters. In the purple cluster, *N. nucifera* , *N. coerulea* , *L. indica* (Pink), *L. indica* (Red), and *P. granatum* were grouped together, reflecting their shared botanical subfamilies. Specifically, *N. nucifera* and *N. caerulea* are members of the *Nymphaeaceae* family, while *L. indica* (Pink) and *L. indica* (Red) belong to the *Lythraceae* family. This observation suggested that edible flowers from the same botanical family tend to share similar active components and antioxidant capacities. Conversely, in the red cluster, *A. africanus* , *S. japonica* , and *H. citrina* were grouped because of their lower content of active ingredients and relatively weaker antioxidant capacities.

#### 3.5.3 Fuzzy Membership Function Analysis

Fuzzy membership function analysis is a mathematical method used to represent fuzzy sets by quantifying the degree of membership for each element, with values ranging from 0 to 1 (Lu et al. 2022). The raw data for each indicator were standardized using SPSS 27 to address potential issues caused by differing dimensions. Principal component analysis was then used to reduce dimensionality and simplify the dataset. Subsequently, fuzzy membership function analysis was used to standardize the 10 quality indicators of the samples. The membership function values for each indicator were calculated using the following formula, and the overall evaluation value was derived on the basis of these values (Zou et al. 2023; Liao et al. 2022).

$$\mu_{\mathrm{ij}}=\left(X_{\mathrm{ij}}-X_{j\min}\right)/\left(X_{j\max}-X_{j\min}\right)$$

$$D=\sum \mu_{\mathrm{ij}}/i$$

where *μ*_*ij*_ represents the membership function value of the *j*th index for the *i*th flower, *X*_*ij*_ represents the measured value of the *j*th index for the *i*th flower, *X*_*j*min_ and *X*_*j*max_ represent the minimum and maximum values of the *j*th index among all flowers, and *D* represents the comprehensive evaluation value of the various indicators for the 50 edible flowers.

The comprehensive evaluation of the 50 edible flowers was conducted using the fuzzy membership function method. The results, presented in Table 6, showed that *P. granatum* achieved the highest comprehensive score, indicating the best overall quality, followed by *L. indica* (Red). In contrast, *A. africanus* and *H. citrina* had the lowest comprehensive scores, reflecting the lowest levels of active components and antioxidant capacity.

**TABLE 6:** The membership function values and rank of 50 edible flowers.

Flower samples	Membership function values	Rank
*Agapanthus africanus*	0.3479	49
*Lycoris radiata* (L'Her.) Herb.	0.4368	43
*Narcissus tazetta* L. var. chinensis Roem.	0.4104	45
*Chimonanthus praecox* (Linn.) Link	0.5119	30
*Platycodon grandiflorus* (Jacq.) A. DC.	0.6198	14
*Canna indica* L.	0.4820	35
*Lonicera japonica* Thunb.	0.5166	28
*Dendranthema morifolium* (Ramat.) Tzvel.	0.5191	27
*Cucurbita moschata* (Duch. ex Lam.) Duch. ex Poiret	0.4135	44
*Rhododendron lapponicum*	0.7096	5
*Gentiana macrophylla* Pall.	0.4076	46
*Gentiana sino‐ornata* Balf. f.	0.5199	26
*Hypericum monogynum* L.	0.5783	17
*Iridis japonica*	0.5825	16
*Iris tectorum*	0.5081	33
*Iris pseudacorus*	0.3837	48
*Clerodendranthus spicatus* (Thunb.) C. Y. Wu	0.5103	31
*Lavandula angustifolia*	0.4550	41
*Albizia julibrissin* Durazz.	0.5247	25
*Clitoria ternatea*	0.5461	21
*Sophora japonica* Linn.	0.4705	38
*Hemerocallis citrina* Baroni	0.3370	50
*Lilium formosanum* Wallace	0.5262	24
*Buddleja officinalis* Maxim.	0.5409	22
*Lagerstroemia indica* L. (Pink)	0.7867	3
*Lagerstroemia indica* L. (Red)	0.7937	2
*Magnolia denudata* Desr. (White)	0.5129	29
*Magnolia denudata* Desr. (Purple)	0.5076	34
*Magnolia liliiflora* Desr	0.5486	20
*Althaea rosea* (Linn.) Cavan.	0.4549	42
*Hibiscus syriacus* Linn. (Double)	0.4647	40
*Hibiscus rosa‐sinensis*	0.6504	11
*Hibiscus syriacus* Linn. (Single)	0.3851	47
*Musa basjoo*	0.4750	37
*Nelumbo nucifera*	0.6669	9
*Nymphaea coerulea*	0.7066	6
*Jasminum nudiflorum* Lindl.	0.5100	32
*Jasminum sambac* (L.) Ait.	0.4703	39
*Osmanthus fragrans* (Thunb.) Lour.	0.6298	12
*Dendrobium officinale* Kimura et Migo	0.4804	36
*Punica granatum* L.	0.8053	1
*Cerasus serrulata*	0.6524	10
*Chaenomeles speciosa*	0.5531	19
*Rosa rugosa* Thunb. (Mohong)	0.6806	7
*Prunus mume*	0.6721	8
*Prunus persica*	0.5344	23
*Rosa damascena*	0.6252	13
*Rosa rugosa* Thunb. (Dianhong)	0.7286	4
*Camellia japonica* L. (Double)	0.5664	18
*Camellia japonica* L. (Single)	0.5962	15

## 4 Discussion

### 4.1 Active Component Analysis

Edible flowers contain phenolic compounds such as phenolic acids, flavonols, and anthocyanins. These compounds contribute to their antioxidant capacity by protecting against damage caused by free radicals and have demonstrated positive effects on human metabolism (Navarro‐González et al. 2014). Phenolics represent a diverse group of aromatic secondary plant metabolites existing in free and conjugated forms. They can be found in soluble and insoluble states and are often bound to sugars, acids, or other biomolecules (Xiong et al. 2014). However, the content and medicinal properties of polyphenols, flavonoids, and anthocyanins in edible flowers remain inadequately characterized (Albert et al. 2023). This study revealed that *P. mume* had the highest TPC, *O. fragrans* had the highest TFC, and *R. rugosa* (Dianhong) exhibited the highest TAC. These results align with those reported by Chen et al. (2015), who assessed the total phenolic and flavonoid contents and antioxidant capacities of 23 edible flowers. In their study, TFC ranged from 0.45 ± 0.01 to 71.49 ± 0.86 mg RE/g, indicating a 158.86‐fold variation. *O. fragrans* demonstrated the highest total flavonoid content, followed by *L. angustifolia* and *R. officinalis*. Comparing active components across various edible flowers remains challenging because of the diversity of flower species and methodological variations among studies, complicating the identification of specific reference flowers.

### 4.2 Antioxidant Activity Analysis

The capacity of natural substances to scavenge free radicals serves as a critical indicator of their antioxidant potential (Nie et al. 2019). However, reliance on a single index may lack specificity and sensitivity, resulting in an incomplete assessment of antioxidant activity in flower samples. In this study, three indices—FRAP values and the scavenging rates of DPPH and ABTS radicals—were employed to assess the antioxidant capacity of 50 edible flowers. The results showed that *R. rugosa* (Mohong) had the strongest DPPH and ABTS radical scavenging activities among the 50 edible flowers investigated, which is consistent with the findings of Xiao et al. (2019). Their study analyzed the polyphenol content and radical scavenging capacity of fresh *R. rugosa* (Mohong) extract using DPPH, ABTS, ˙OH, and FRAP assays. The results demonstrated significant in vitro antioxidant activity, supporting its potential for use in natural antioxidant applications. Furthermore, *R. rugosa* (Dianhong) and *R. rugosa* (Mohong), two important edible rose cultivars from Yunnan, demonstrated significant antioxidant activity. Notably, *R. rugosa* (Dianhong) had the highest TAC among the 50 flowers analyzed. These findings highlight the potential of edible flowers as promising candidates for further exploration, particularly in the context of dietary interventions aimed at mitigating oxidative stress and related chronic diseases. Therefore, edible flowers represent valuable sources of bioactive compounds in the human diet.

Evaluating flower quality on the basis of a single nutrient or antioxidant index lacks scientific robustness and may introduce bias. Therefore, a comprehensive quality evaluation method is crucial (Jin et al. 2022). Pearson correlation analysis, a widely used statistical method, is effective for assessing the strength of relationships between variables. Zhang, Cao, et al. (2023) used Pearson correlation analysis to examine the nutritional composition and antioxidant capacities of 34 edible flowers. Their results revealed significant positive correlations (*p* < 0.05) between TPC and FRAP values, DPPH scavenging rates, and ABTS scavenging rates, with the highest correlation (*r* = 0.66) observed between TPC and ABTS scavenging. These findings align with those of Rajurkar and Hande (2011), who also reported a strong correlation between TPC and ABTS scavenging capacity. These results indicate that the antioxidant capacity of edible flowers is closely linked to their polyphenol content. Additionally, cluster analysis showed that edible flowers within the same botanical family tend to exhibit similar active components and antioxidant capacities. Membership function analysis further identified that *N. coerulea* , *R. rugosa* , *P. granatum* , and *L. indica* achieved the highest comprehensive scores, demonstrating superior overall quality. These flowers emerge as notable sources of natural antioxidants and antimicrobial agents.

### 4.3 Antimicrobial Activity In Vitro Analysis

Studies have demonstrated that extracts from five edible flowers, including *N. coerulea* , *L. indica* (Red), *P. granatum* , *L. japonica* , and *L. indica* (Pink), exhibit significant antimicrobial effects against *E. coli* . *N. coerulea* , a member of the *Nymphaeaceae* family, is known for its diverse pharmacological properties, including calming, pain relief, moisturizing, whitening, free radical scavenging activities, and the ability to lower cholesterol, blood sugar, and blood pressure. This study confirmed that *N. coerulea* exhibited significant antimicrobial activity against *E. coli* and *S. aureus* , with inhibition zones of 12.11 ± 0.85 mm and 12.42 ± 0.61 mm, respectively, indicating moderate sensitivity. These results align with previous research suggesting that components such as gallotannic acid and ellagic acid contribute to its antibacterial properties (Dosoky et al. 2023). In addition to *N. coerulea* , extracts from eight edible flowers, including *L. radiata* , *L. indica* (Pink), *L. indica* (Red), and *P. granatum* , also exhibited significant antimicrobial effects against *S. aureus* . *L. indica* , a member of the *Lythraceae* family, is commonly referred to as Indian crape myrtle or crepe flower. It is native to regions such as China, Korea, Japan, India, and the United States, where it is often cultivated as an ornamental shrub (Wei and Liu 2022). The study by Wei reported that pink, mauve, and carmine flowers exhibited antimicrobial activity against *S. aureus* , *P. aeruginosa* , *E. coli* , and *B. subtilis* , with an MIC value of 0.078 mg/mL. Furthermore, extracts from two additional edible flowers, *H. rosa‐sinensis* and *P. grandiflorus* , demonstrated excellent antimicrobial effects against *P. aeruginosa* .

This study suggests that a direct correlation does not always exist between the agar diffusion method, which measures inhibition zones, and the microdilution method used for determining MIC and MBC. Discrepancies between these methods may arise from the presence of multiple bioactive components in medicinal plant extracts, each exhibiting varying potency levels (Alzoreky and Nakahara 2003). Moreover, differences in the diffusion rates of these components in agar can affect inhibition zone formation, a factor not accounted for in MIC determination. Additionally, the inherent color of some plant extracts can interfere with the visual assessment of turbidity during MIC testing in liquid media. Therefore, the agar diffusion method alone is inadequate for a comprehensive evaluation of the antibacterial activity of medicinal plant extracts. A comprehensive approach that includes MIC and MBC determinations is essential. Future research should focus on identifying and isolating individual bioactive components, conducting parallel experiments, and refining MIC and MBC measurements to enhance accuracy and comprehensiveness. This study primarily focused on evaluating the in vitro antibacterial activity of medicinal plant extracts, representing only one aspect of their broader characteristics. Further investigation is needed to elucidate the mechanisms of action and assess the in vivo efficacy of these extracts in order to better understand their therapeutic applications.

## 5 Conclusions

This study assessed the active components, antioxidant capacities, TYR inhibition rates, and in vitro antimicrobial activities of 50 edible flowers from Yunnan Province. The results showed that *P. mume* had a high TPC, whereas *O. fragrans* had the highest TFC. *R. rugosa* (Dianhong) showed the highest TAC. Among the samples, *R. rugosa* (Mohong) demonstrated the strongest DPPH and ABTS radical scavenging capacities, and *C. spicatus* demonstrated the highest FRAP activity. In terms of TYR inhibition, *P. granatum* showed the highest activity. *N. coerulea* exhibited significant antimicrobial effects against *E. coli* and *S. aureus* , and extracts from *H. rosa‐sinensis* and *P. grandiflorus* demonstrated excellent antimicrobial effects against *P. aeruginosa* . Correlation analysis indicated significant positive relationships between TPC and FRAP values, DPPH scavenging rates, and ABTS scavenging rates, suggesting that antioxidant capacity is closely associated with polyphenol content. This study provides valuable insights into the potential of 50 edible flowers as sources of natural antioxidants and antimicrobial agents. Membership function analysis further identified *N. coerulea* , *R. rugosa* , *P. granatum* , and *L. indica* as having the highest comprehensive scores, underscoring their superior overall quality and values as sources of bioactive compounds.

## Author Contributions

**Yanxia Tao:** conceptualization (equal), methodology (equal), validation (equal), writing – original draft (equal), writing – review and editing (equal). **Xingkai Zhang:** conceptualization (equal), data curation (equal), supervision (equal). **Zhuangyu Li:** methodology (equal), writing – original draft (equal). **Shouzheng Tian:** data curation (equal), funding acquisition (equal). **Li Li:** funding acquisition (equal), investigation (equal), writing – review and editing (equal). **Xuhong Zhou:** formal analysis (equal), funding acquisition (equal), supervision (equal), validation (equal), writing – review and editing (equal).

## Ethics Statement

The authors have nothing to report.

## Consent

Written informed consent was obtained from all study participants.

## Conflicts of Interest

The authors declare no conflicts of interest.

## Supporting information


Data S1.

## Data Availability

Data are contained within the article or Supporting Information.
