# Supplementary material for: Comprehensive Analysis of 50 Edible Flowers From Yunnan Province: Active Components, Antioxidant Capacity, Tyrosinase Inhibition, and Antimicrobial Activity
Source: Food Sci Nutr. 2025 Jul 21;13(7):e70666. doi: 10.1002/fsn3.70666 (PMC12279552; doi:10.1002/fsn3.70666)
Supplement: Supplementary file 1 — Data S1. [file FSN3-13-e70666-s001.docx]

**SUPPLEMENT |** Ferric reducing antioxidant power and tyrosinase inhibition activity of the 50 edible flowers.

| **No.** | **Flower Samples** | **FRAP (mmol/L)** | **TYR (%)** |
| --- | --- | --- | --- |
| 1 | *Agapanthus africanus* | 0.09 ± 0.00 | 9.44 ± 3.02 |
| 2 | *Lycoris radiata* (L’Her.) Herb. | 0.07 ± 0.01 | - |
| 3 | *Narcissus tazetta* L. var. chinensis Roem. | 0.11 ± 0.00 | 3.49 ± 1.69 |
| 4 | *Chimonanthus praecox* (Linn.) Link | 0.50 ± 0.01 | 21.91 ± 0.45 |
| 5 | *Platycodon grandiflorus* (Jacq.) A. DC. | 0.09 ± 0.00 | 5.86 ± 1.87 |
| 6 | *Canna indica* L. | 0.45 ± 0.01 | - |
| 7 | *Lonicera japonica* Thunb. | 0.28 ± 0.01 | 12.11 ± 2.53 |
| 8 | *Dendranthema morifolium* (Ramat.) Tzvel. | 0.51 ± 0.01 | 7.62 ± 2.56 |
| 9 | *Cucurbita moschata* (Duch. ex Lam.) Duch. ex Poiret | 0.04 ± 0.00 | 15.76 ± 4.73 |
| 10 | *Rhododendron lapponicum* | 0.80 ± 0.01 | 43.94 ± 4.20 |
| 11 | *Gentiana macrophylla* Pall. | 0.12 ± 0.00 | 7.99 ± 3.63 |
| 12 | *Gentiana sino-ornata* Balf. f. | 0.31 ± 0.00 | 14.11 ± 2.41 |
| 13 | *Hypericum monogynum* L. | 0.55 ± 0.02 | - |
| 14 | *Iridis Japonica* | 0.07 ± 0.00 | 15.96 ± 2.33 |
| 15 | *Iris tectorum* | 0.09 ± 0.00 | 27.68 ± 2.58 |
| 16 | *Iris pseudacorus* | 0.08 ± 0.00 | 15.46 ± 3.37 |
| 17 | *Clerodendranthus spicatus* (Thunb.) C. Y. Wu | 1.13 ± 0.00 | - |
| 18 | *Lavandula angustifolia* | 0.70 ± 0.04 | - |
| 19 | *Albizia julibrissin* Durazz. | 0.20 ± 0.01 | 12.20 ± 2.23 |
| 20 | *Clitoria ternatea* | 0.14 ± 0.00 | 11.81 ± 0.98 |
| 21 | *Sophora japonica* Linn. | 0.06 ± 0.00 | 14.54 ± 2.75 |
| 22 | *Hemerocallis citrina* Baroni | 0.03 ± 0.00 | - |
| 23 | *Lilium formosanum* Wallace | 0.18 ± 0.00 | 15.04 ± 3.34 |
| 24 | *Buddleja officinalis* Maxim. | 0.64 ± 0.01 | - |
| 25 | *Lagerstroemia indica* L. (Pink) | 0.94 ± 0.02 | 55.58 ± 2.32 |
| 26 | *Lagerstroemia indica* L. (Red) | 0.76 ± 0.02 | 67.25 ± 1.79 |
| 27 | *Magnolia denudata* Desr. (White) | 0.59 ± 0.02 | - |
| 28 | *Magnolia denudata* Desr. (Purple) | 0.31 ± 0.02 | - |
| 29 | *Magnolia liliiflora* Desr | 0.75 ± 0.01 | - |
| 30 | *Althaea rosea* (Linn.) Cavan. | 0.19 ± 0.00 | - |
| 31 | *Hibiscus syriacus* Linn. (Double) | 0.09 ± 0.00 | 10.71 ± 2.91 |
| 32 | *Hibiscus* *rosa-sinensis* | 0.33 ± 0.00 | - |
| 33 | *Hibiscus syriacus* Linn. (Single) | 0.09 ± 0.00 | 4.37 ± 2.48 |
| 34 | *Musa basjoo* | 0.27 ± 0.00 | 15.37 ± 7.59 |
| 35 | *Nelumbo nucifera* | 1.04 ± 0.02 | 54.76 ± 4.99 |
| 36 | *Nymphaea coerulea* | 0.96 ± 0.06 | 50.50 ± 2.93 |
| 37 | *Jasminum nudiflorum* Lindl*.* | 0.16 ± 0.00 | 3.27 ± 0.96 |
| 38 | *Jasminum sambac* (L.) Ait. | 0.20 ± 0.01 | 11.27 ± 3.40 |
| 39 | *Osmanthus fragrans* (Thunb.) Lour. | 0.97 ± 0.00 | - |
| 40 | *Dendrobium officinale* Kimura et Migo | 0.11 ± 0.00 | 9.65 ± 3.49 |
| 41 | *Punica granatum* L. | 0.73 ± 0.01 | 73.76 ± 0.76 |
| 42 | *Cerasus serrulata* | 0.46 ± 0.01 | 11.53 ± 3.76 |
| 43 | *Chaenomeles speciosa* | 0.14 ± 0.00 | - |
| 44 | *Rosa rugosa* Thunb. (Mohong) | 0.91 ± 0.03 | 66.23 ± 5.23 |
| 45 | *Prunus mume* | 0.57 ± 0.00 | - |
| 46 | *Prunus persica* | 0.20 ± 0.00 | - |
| 47 | *Rosa damascena* | 0.89 ± 0.01 | 46.22 ± 2.25 |
| 48 | *Rosa rugosa* Thunb. (Dianhong) | 0.75 ± 0.01 | 47.21 ± 6.01 |
| 49 | *Camellia japonica* L. (Double) | 0.38 ± 0.00 | - |
| 50 | *Camellia japonica* L. (Single) | 0.86 ± 0.02 | - |

“-” stands for edible flowers have not Ferric Reducing Antioxidant Power/ tyrosinase inhibition activity. The table presents the optimal results for Ferric Reducing Antioxidant Power and tyrosinase inhibition activity for each flower.
